# Knowledge, Attitudes, and Practices toward First Aid Management of Skin Burns in Saudi Arabia

**DOI:** 10.3390/clinpract12010013

**Published:** 2022-02-15

**Authors:** Mahdi Al Dhafiri, Feroze Kaliyadan, Mohammed A. Alghadeer, Zainab Y. AL-Jaziri, Zainab A. Alabdulmuhsin, Zainab A. Alaithan

**Affiliations:** 1 Division of Dermatology, Department of Internal Medicine, College of Medicine, King Faisal University, Al-Ahsa 31982, Saudi Arabia; 2 Department of Dermatology, Sree Narayana Institute of Medical Sciences, Ernakulam 683101, India; ferozkal@hotmail.com; 3 Department of Surgery, King Faisal Hospital, Al-Ahsa 31982, Saudi Arabia; mohammed.abdulmajeed.gh@hotmail.com; 4 Medical Intern, College of Medicine, King Faisal University, Al-Ahsa 31982, Saudi Arabia; z.jaziri97@gmail.com (Z.Y.A.-J.); zainab.112@gmail.com (Z.A.A.); vovo2442@hotmail.com (Z.A.A.)

**Keywords:** burns, a cross-sectional study, first aid management of burns, Eastern province of Saudi Arabia

## Abstract

**Background:** Burns are considered a serious health affection that leads to several consequences affecting a person both physically and emotionally. Herbal and traditional medicine have become popular remedies among patients worldwide. **Aim:** This study explores common practices followed in burns as first aid management. **Methods:** A cross-sectional study was conducted in the Eastern province of Saudi Arabia using a designed questionnaire distributed electronically through different social media. The questionnaire consisted of socio-demographic details, history of burns, causative material, and practices followed in response to burns. **Results:** 461 individuals have participated in this study. The commonest reason for burns was hot water or steam. The majority of the respondents (63%) had a satisfactory response to self-aid alone at home. The most common first aid options for managing burns at home were cold water alone 195 (42%), any sort of cream alone 177 (38%), or both 317 (69%). Overall, the result shows no statistically significant difference between the outcome of burn injury and the most commonly used burn aids. **Conclusions:** Most people use creams and water as the first-aid management of burns, while a good number of people use traditional medicine. Overall, people who receive hospital treatment after getting first aid at home give a better outcome.

## 1. Introduction

Burns are defined as any injury or damage to the human organic tissue, such as skin, primarily attributed to heat exposure in the form of scalds and flame. Radioactivity, electricity, friction, and chemical contacts are categorized as secondary attributions [1].

Burn wounds cause high levels of morbidity and mortality worldwide. People with burns are particularly vulnerable to infections; over 75% of all burn deaths (after initial resuscitation) result from infection [2]. Antiseptics are topical agents that act to prevent the growth of micro-organisms. A wide range is used to prevent infection and promote the healing of burn wounds [1].

Skin burn injuries cause complex problems, including scar marks, psychological effects, and the affected person’s overall life quality (LQ) [3]. Burns at the exposed areas of the body were more likely to cause emotional and social distress [4]. Several studies have been done on the scar-specific quality of life from the patient’s perspective [4]. There may be different distressing factors associated with psychological problems in patients with burns [5].

The WHO defines traditional medicine as “the sum total of the knowledge, skill, and practices based on theories, beliefs, and experiences indigenous to different cultures, whether explicable or not, used in the maintenance of health as well as in the prevention, diagnosis, improvement or treatment of physical and mental illness” [6]. Depending on the severity of the burn and the depth of the damage, medical interventions vary. There is a continuous tendency to use traditional medicine as alternative treatments in minor burns [7]. It has become a popular treatment among patients with various diseases worldwide [8]. A study published in Saudi Arabia revealed a high prevalence of herbal medicine use among patients with chronic diseases [9]. The purpose of our study is to evaluate the different traditional therapeutic interventions for skin burns and their outcome and the population’s belief and knowledge toward them in Saudi Arabia.

## 2. Methodology

After obtaining the Institutional Review Board Approval from King Fahad Hospital, Hufof, Saudi Arabia. A cross-sectional study was conducted among the eastern province of the Saudi population between 27 January 2021 and 10 February 2021. Data was collected through an online survey validated by two dermatologists, prepared via Google forms, and distributed through electronic social media, including WhatsApp, Twitter, and Telegram applications. A pilot study was conducted among 24 subjects, which were excluded from the final result. Participants who met the inclusion criteria of being Saudi and had a history of burns within the past five years were selected. On the other hand, non-Saudi participants, or those who did not have a burn in the last five years were excluded from the study. After clarifying the study’s purpose and obtaining online written consents from the participants, 461 participants addressed the conducted questionnaire. The questionnaire included three sections; the first was about personal information, including age, gender, social status, education level, work, and type of housing. The second section was about the exposure to burn, the cause of the burn, the affected part of the body, and the grade of burn. The third section was about their attitude towards the burn, which included first aid, substance applied on the burned area, knowledge source about the management, duration of healing, and the estimated number of burns per year among family members.

The collected data was entered and analyzed using the Statistical Package for the Social Science (SPSS Inc., Chicago, IL, USA) version 26. Descriptive statistics were performed. Percentages were given for qualitative variables. The determinant factors were determined using the Chi-square test. *p*-value was considered significant if *p* < 0.05.

## 3. Results

There were 461 valid responses (27% male and 73% female) (Table 1). The age ranged from 11 to 70 years, with a mean of 33.14 years. The level of education was university or higher in 65.4% of the respondents. Among the respondents, 62.8% were married, 59.7% were living in a house, 62.6% were not employed, and 47.6% had no children.

The commonest source of burns was hot water or steam (174 (38%)), followed by contact with a hot metal (147 (32%)). Hot oil and direct contact with a flame were the other common sources of burns. Hands were the commonest affected site (309 (67%)). The majority of the cases were seen to have first-degree burns with superficial to superficial partial-thickness burns (erythema without blisters (189 (41%)), second degree burn with erythema, and blistering (248 (54%)). Third-degree with deeper burns were reported in (54 (12%)) respondents.

The majority of the respondents had a satisfactory response to self-aid alone at home (366 (79%)), while the rest had to go to the hospital for medical care. The commonest self-administered aid used included over-the-counter creams/ointments like Moist Exposed Burn Ointment (MEBO^®^), which 52 (12%) of the respondents mentioned. Other self-aid measures tried to include cold water, honey, flour, starch, and ice.

The most common first aids option for managing burns at home was cold water alone (195 (42%)), any cream for burns alone (177 (38%)), or both cold water and cream (317 (69%)).

The sources of information for self-administered aid (for those not working in the health care field) were mainly the internet and social media. Most of the participants (75.1%) dealt with the burn injury at home without going to the hospital, and 15.1%. Only 24.9% went to the hospital directly.

Complete improvement of the burn was seen in 233 (51%) of the respondents, while complications were seen in some respondents like hyperpigmentation (68 (15%)), atrophy (36 (8%)), and hypopigmentation (53 (11%)). The other patients had a combination of hyperpigmentation/hypopigmentation and atrophy. Of the total, 71 (16%) respondents had prolonged healing time (more than one month), while a majority (326 (71%)) healed completely in a month, of which 197 had healed within two weeks (Table 2).

Concerning first-degree burns, the results of our study show no statistically significant relations between the outcome of burn injury and the most commonly used burn aids (Table 3).

According to the second-degree burns, there was a statistically significant relation between using MEBO^®^ cream as burn treatment and the outcome of the burn injury. However, the results show no statistically significant relations between the outcome of burn injury and the other most used burn aids (Table 4).

Regarding third-degree burns, the results of our study show no statistically significant relations between the outcome of burn injury and the most commonly used burn aids (Table 5).

## 4. Discussion

Burns are the fourth most prevalent form of trauma worldwide and are one of the most devastating traumatic conditions confronted in practice, affecting patients’ physical and psychological state at all ages. [10]. Annually, burns result in more than 7.1 million injuries and more than 250,000 deaths worldwide [11].

After a minor burn injury, promptly applying first aid plays a vital role in determining the outcome and level of comorbidity and limiting tissue damage, including the need for surgery [12]. Several studies have found a lack of widespread knowledge of burn first aid among both general population members and professional healthcare providers [13].

The present study indicated that electrical burns were reported in 1.8% and chemical burns in 1.6%. Almarghoub et al. [14], in a systematic review that summarized results on the epidemiology of burn injuries in Saudi Arabia, reported that Scald injuries (hot water or steam) accounted for 62.4% of injuries, followed by flame-induced burns (28.7%), electrical burns (3.3%), and chemical burns (2.8%). Almost 60% were second-degree burns.

Our results showed that the majority of respondents had dealt with burn injuries (either personally or by helping another person) in the past five years (N = 461, 93.3%). The commonest causes of burn were scalding burns 38.4% (contact hot water 25.6% or hot steam 12.8%), contact with a hot metal such as an oven, saucepan, or pipe (31.5%), hot oil (15%), and contact with a flame such as fire, charcoals, or firewood (10.8%).

A cross-sectional study conducted by Batais et al. [15] in Riyadh, Saudi Arabia, on medical and non-medical university students to determine knowledge and practice of burn first aids showed that 61.8% reported having experienced a burn injury personally or in a close family member. Of these, 54.4% reported that they administered or received first aid for a burn. Treatments reported included 81.8% having applied water to the injured area, 72.3% having removed clothing or accessories from the area of injury, and 71 (51.8%) using ice to cool the injured area. Another study from Pakistan in Rawalpindi showed that toothpaste (47.5%) followed by cool running water (20.3%) were the two most frequently applied items following a burn injury [16], while in Nigeria, water lavage was used in 49 (29.2%) cases, raw eggs in 21 (12.5%), pap in 16 (9.5%) [17]. Similar to our findings, another study conducted in Saudi Arabia by Almutlaq et al. 2020 [5] reported that most respondents used cold water or honey as post-burn therapy. Though studies showed that using honey does not effectively reduce the extent of burn of the affected tissue, recently, it has been reported that silver sulfadiazine, along with honey, is effective in burn wound healing [18]. Most studies have shown a lack of knowledge of burn first aid in groups such as medical students and healthcare workers in the United Kingdom, the Saudi population, students in Nigeria, and adults in Australia [19]. In Saudi pediatric health care professionals, Alomar et al. 2016 [20] found that only 41% were aware of using cold water as an aid to burn wounds, only 15% had burn first-aid training, and only 3% knew the appropriate duration of cold-water use. For the present study, among those who did not go directly to the hospital and received first aid at home (94.1%), the most common burn aids locally used on burn site were moist exposed burn ointment (MEBO^®^) (42.9%), and cold water (41%). Others include creams for burns (24.2%), flour (22.1%), ice (21.2%), lukewarm water (20.0%), honey (13.1%), cactus gel (3.5%), and starch (3%). The least commonly used was toothpaste (1.84%).

Unlike Fadeybi et al., 2015 [17], who reported that patients that received no water first aid had a higher complication rate (35.3% versus 18.4%) compared with those that had water first aid, our results showed no statistically significant relation between complete recovery of burn injury and having used cold water or MEBO^®^ cream as first aid. However, unlike our study’s population, Fadeybi et al. recruited the admitted patients in the burn unit who could have more severe burn conditions. These results are compatible with a systematic review that concluded that the evidence for MEBO^®^ in English-language literature was poor and inconsistent with wound healing rate and analgesia compared to 1% Silver Sulfadiazine Cream 1% (SSD 1%), Acquacel Ag, *Helix aspersa* cream, and povidone-iodine + panthenol cream.

One of the earliest records of burn treatments in Egypt advocated the use of resin and honey salve in 1600 BC. The Chinese subsequently described the use of tea leaves in 600 BC. Hippocrates described the use of bulky dressings with pig fat, resins, warm vinegar soaks, and tanning solutions made from oak bark. The use of cold water for treating burns was not described until 854 CE by an Arabian physician [21].

While several studies have postulated mechanisms for which traditional remedies may be beneficial, there is a paucity of evidence to support their use, and clinical reports are often anecdotal. Some groups have attempted to evaluate the pharmacological properties and mechanisms of action of a number of traditional remedies, including SulconaW, a traditional Siddha medicine originating from Southern India [22], and the mixture of olive oil and lime cream originating from Turkey [23]. The Amish community has been observed using traditional remedies such as a combination of burns and wounds ointment (typically aloe vera, lanolin, honey, white oak bark, comfrey root, and lobelia) with burdock leaves [24].

In our results, the source of information about the used aid was a family member or friend in 62% of cases, social media in 25.8%, only 22.6% were doctors/nurses or were advised by a pharmacist. Al-Johani et al. 2018 [25] in Madinah, Saudi Arabia, in a similar study, stated that the commonest reported source of information among parents about first aid was social media (59%), followed by schoolbooks (14.9%). Only 13% of them reported that the source of their information was doctors or nurses.

Our results also showed that 47.2% of cases have fully recovered, while 31.2% showed hyperpigmentation/hypopigmentation. Time of healing was one to two weeks in 39.2%.

## 5. Conclusions

With its different degrees, people respond differently to burns. Different home remedies and over-the-counter creams are used as first aid management in case of skin burn. Most people use creams as first aid management of burns, while a good number of people use traditional medicine. The non-medical sources of information, including family members, friends, and social media, play a major role in these practices. Thus, the level of education and the source of information plays a major role in that. Overall, people who receive hospital treatment after getting first aid at home give a better outcome. Increasing the education and awareness towards first-aid intervention in skin burns plays a crucial role in getting a better outcome.

## 6. Limitations

Our study depends on a self-conducted electronic survey, and the risk of bias regarding the burn’s degree and healing durations is not negligible.

Additionally, the duration of the usage of traditional remedies until reaching the healing was not conducted.

## Figures and Tables

**Table 1:** Participants who dealt with burn injuries in the last five years, Al Ahsa, KSA, 2021 (N = 461).

Treated by:	Responses (N = 461)
Self-aid	292 (63%)
A friend	24 (5%)
Son or daughter	50 (11%)
Another family member	95 (21%)

**Table 2:** Healing Time for participants who dealt with burn injuries in the last five years using traditional medicine, Al Ahsa, KSA, 2021 (N = 434).

Healing Time for Traditional Medicine:	Responses (N = 434)
1–3 Days	91 (21%)
4–6 Days	62 (14%)
1–2 weeks	170 (39%)
3–4 weeks	51 (12%)
More than one month	60 (14%)

**Table 3:** Shows the relation between each commonly used burn aid and the outcome of the burn injury among participants with a 1st-degree burn. (N = 168).

Remedy Used		Outcome				Total (N = 168)	*p* Value
		Burn Improvement (Full Recovery)	Hyperpigmentation/ Hypopigmentation	Skin Atrophy	Inflammation/Skin Hypertrophy		
cold water	Yes	40	19	8	3	70	0.526
		57.1%	27.1%	11.4%	4.3%	100.0%	
	No	58	30	9	1	98	
		59.2%	30.6%	9.2%	1.0%	100.0%	
MEBO^®^ cream	Yes	31	21	7	1	60	0.528
		51.7%	35.0%	11.7%	1.7%	100.0%	
	No	67	28	10	3	108	
		62.0%	25.9%	9.3%	2.8%	100.0%	
warm water	Yes	22	6	1	2	31	0.081
		71.0%	19.4%	3.2%	6.5%	100.0%	
	No	76	43	16	2	137	
		55.5%	31.4%	11.7%	1.5%	100.0%	
other creams for burns	Yes	23	10	7	1	41	0.382
		56.1%	24.4%	17.1%	2.4%	100.0%	
	No	75	39	10	3	127	
		59.1%	30.7%	7.9%	2.4%	100.0%	
ice	Yes	19	11	4	3	37	0.073
		51.4%	29.7%	10.8%	8.1%	100.0%	
	No	79	38	13	1	131	
		60.3%	29.0%	9.9%	0.8%	100.0%	
honey	Yes	10	7	5	0	22	0.147
		45.5%	31.8%	22.7%	0.0%	100.0%	
	No	88	42	12	4	146	
		60.3%	28.8%	8.2%	2.7%	100.0%	
flour	Yes	16	11	6	0	33	0.208
		48.5%	33.3%	18.2%	0.0%	100.0%	
	No	82	38	11	4	135	
		60.7%	28.1%	8.1%	3.0%	100.0%	

**Table 4:** Relation between each commonly used burn aid and the outcome of the burn injury among participants with a 2nd-degree burn. (N = 243).

Remedy Used		Outcome				Total (N = 243)	*p* Value
		Burn Improvement (Full Recovery)	Hyperpigmentation/ Hypopigmentation	Skin Atrophy	Inflammation/Skin Hypertrophy		
cold water	Yes	43	29	10	10	92	0.324
		46.7%	31.5%	10.9%	10.9%	100.0%	
	No	60	58	23	10	151	
		39.7%	38.4%	15.2%	6.6%	100.0%	
MEBO^®^ cream	Yes	47	33	21	4	105	0.011
		44.8%	31.4%	20.0%	3.8%	100.0%	
	No	56	54	12	16	138	
		40.6%	39.1%	8.7%	11.6%	100.0%	
warm water	Yes	22	17	7	4	50	0.991
		44.0%	34.0%	14.0%	8.0%	100.0%	
	No	81	70	26	16	193	
		42.0%	36.3%	13.5%	8.3%	100.0%	
other creams for burns	Yes	22	22	7	0	51	0.098
		43.1%	43.1%	13.7%	0.0%	100.0%	
	No	81	65	26	20	192	
		42.2%	33.9%	13.5%	10.4%	100.0%	
ice	Yes	23	15	11	3	52	0.238
		44.2%	28.8%	21.2%	5.8%	100.0%	
	No	80	72	22	17	191	
		41.9%	37.7%	11.5%	8.9%	100.0%	
honey	Yes	12	7	8	2	29	0.107
		41.4%	24.1%	27.6%	6.9%	100.0%	
	No	91	80	25	18	214	
		42.5%	37.4%	11.7%	8.4%	100.0%	
flour	Yes	23	24	10	2	59	0.308
		39.0%	40.7%	16.9%	3.4%	100.0%	
	No	80	63	23	18	184	
		43.5%	34.2%	12.5%	9.8%	100.0%	

**Table 5:** Relationship between each commonly used burn aid and the outcome of the burn injury among participants with a 3rd-degree burn. (N = 50).

Remedy Used		Outcome				Total (N = 50)	*p* Value
		Burn Improvement (Full Recovery)	Hyperpigmentation/ Hypopigmentation	Skin Atrophy	Inflammation/Skin Hypertrophy		
cold water	Yes	6	5	7	3	21	0.582
		28.6%	23.8%	33.3%	14.3%	100.0%	
	No	11	4	7	7	29	
		37.9%	13.8%	24.1%	24.1%	100.0%	
MEBO^®^ cream	Yes	7	6	6	3	22	0.434
		31.8%	27.3%	27.3%	13.6%	100.0%	
	No	10	3	8	7	28	
		35.7%	10.7%	28.6%	25.0%	100.0%	
warm water	Yes	3	1	2	3	9	0.706
		33.3%	11.1%	22.2%	33.3%	100.0%	
	No	14	8	12	7	41	
		34.1%	19.5%	29.3%	17.1%	100.0%	
cream for burns	Yes	8	2	2	2	14	0.184
		57.1%	14.3%	14.3%	14.3%	100.0%	
	No	9	7	12	8	36	
		25.0%	19.4%	33.3%	22.2%	100.0%	
ice	Yes	4	2	3	0	9	0.429
		44.4%	22.2%	33.3%	0.0%	100.0%	
	No	13	7	11	10	41	
		31.7%	17.1%	26.8%	24.4%	100.0%	
honey	Yes	3	0	3	1	7	0.489
		42.9%	0.0%	42.9%	14.3%	100.0%	
	No	14	9	11	9	43	
		32.6%	20.9%	25.6%	20.9%	100.0%	
flour	Yes	4	0	3	2	9	0.481
		44.4%	0.0%	33.3%	22.2%	100.0%	
	No	13	9	11	8	41	
		31.7%	22.0%	26.8%	19.5%	100.0%	

## Data Availability

All data generated and analyzed during the study are available, and will be provided/ link will be generated to the respected editor upon request.
